# Key Drivers of COVID-19 Vaccine Hesitancy: A Perspective of Collectivism

**DOI:** 10.3390/healthcare11020176

**Published:** 2023-01-06

**Authors:** Yi-Chih Lee, Wei-Li Wu

**Affiliations:** Department of International Business, Chien Hsin University of Science and Technology, Taoyuan 320678, Taiwan

**Keywords:** vaccinations, COVID-19, collectivism, anxiety, vaccine brand loyalty, vaccine hesitancy

## Abstract

Vaccination against the COVID-19 pandemic remains a major part of global immunization policy. The aim of this study was to explore young people’s willingness to continue to receive vaccination against COVID-19 in a collectivist culture. In this study, an online questionnaire was used to measure willingness to continue vaccination, the tendency towards collectivism, the degree of disease anxiety, vaccine brand loyalty, and perceived infectability in 2022. The results showed that women were more willing to be vaccinated than men (70.1% vs. 29.9%). Young people who were willing to receive continuous vaccination had a relatively higher tendency towards collectivism (*p* < 0.001), a relatively higher degree of disease anxiety (*p* < 0.001), and lower vaccine brand loyalty (*p* = 0.034). The COVID-19 pandemic is still ongoing and, since young people are the most active in group activities, policy-makers should weigh the factors influencing vaccination among the young to create effective policy measures.

## 1. Introduction

The world is still being affected by COVID-19. The disease has caused numerous crises in both economics and health for countries around the globe. Vaccines work by training the human body’s immune system to recognize and fight against specified viruses or bacteria. After vaccination, if the body is exposed to the pathogen, the immune system immediately begins to destroy the virus so as to prevent disease. Thus, vaccines can save countless lives [1]. Financial issues, social isolation, lockdown, fear of contagion, and death have had significant effects on mental health during the COVID-19 pandemic. People who often worry about COVID-19 and related issues have an increased likelihood of developing anxiety symptoms [2,3].

Among the different social behaviors, cultural differences are the most frequently raised issues in society. Cultural differences involve the relative emphasis of individualism and collectivism. In individualistic cultures, most people’s social behavior is largely determined by personal goals, attitudes, and the values of society. In collectivist cultures, most people’s social behavior is largely determined by goals, attitudes, and values shared with some group [4]. Hofstede’s (1980) cross-cultural research shows that the West is influenced by individualism [5]; individuals have obvious and fixed boundaries between themselves and others, and that distinction is emphasized in Western society. Individualists only pursue the well-being of themselves and their nuclear family, take individual interests as their starting point, stress individual preference and independence, and place more emphasis on individual characteristics than group ones. Chinese society is defined by collectivism, which takes the protection and promotion of group interests as the principle of social action. Members of the society expect to be accepted by the group, care about the well-being of the group, and emphasize social harmony. Individuals act in accordance with group norms, emphasize group orientation and interests, and place collective interests far above individual interests [6,7]. The Chinese people care about their interactions with other people in daily life, and they are quite concerned about the feelings of others [8]. A collectivist culture attaches great importance to the relationships between members of a group and emphasizes the contribution of individuals to the group. When individual goals are in conflict with group goals, individuals in collectivist cultures will give priority to group goals, while individuals in individualistic cultures will prioritize individual goals [9]. Ting-Toomey and Dorjee (2018) also point out that the “we” identity is more important than the “I” identity, that group rights outweigh individual rights, and that group needs outweigh individual needs and desires in collectivist cultures [10]. Similar to the power distance of cultural values, when people hold collectivist values this also has an effect on willingness to be vaccinated. When people hold strong collectivist values, they care more about the well-being of the group and the impact of illness on others. Therefore, being vaccinated to protect themselves, to avoid harming others, and to protect the health of others becomes an important element of any interpersonal relationship.

In Asian society, individuals follow and conform to the requirements of the social environment, which differs from Western society’s assumption that individual needs should be prioritized [7]. In the context of the pandemic, Chinese society emphasizes that individuals should view themselves in the context of a group. In doing so, individuals must sacrifice their own interests to safeguard public interests and give others the same consideration that they give themselves [11]. Therefore, to avoid the transmission of disease to families or friends, pandemic prevention regulations and initiatives, such as wearing masks and being vaccinated, should be observed. This concept is generally accepted and observed by the Chinese public. According to a survey on willingness to be vaccinated, China is the country with the most support for COVID-19 vaccination [12]. In the United States, it was noted that young people and ethnic minorities have the lowest vaccination rates and willingness to be vaccinated, with an estimated 59.8% of adults aged 18 to 24 being fully vaccinated, compared with 79.0% and 91.5% of adults aged 50 and 64 being vaccinated during the same period [13]. In Taiwan, as of 5 December 2022, the vaccination rate for the COVID-19 vaccine for those over the age of 5 is 94% for the first dose; 88.6% for the second dose; and 74.3% for the booster dose. Taiwanese youth have the highest rate of vaccination against COVID-19 [14].

The World Health Organization (WHO) has defined vaccine hesitancy as the reluctance or refusal to be vaccinated, despite the availability of vaccines. Vaccine hesitancy could contribute to a resurgence of the disease. The reasons for vaccine hesitancy vary, and the WHO maintains that a lack of confidence in vaccines, lack of access to vaccines, or vaccine complacency can contribute to vaccine hesitancy [15]. Furthermore, socioeconomic or healthcare inequalities, rumors and conspiracy theories, and the lack of effective public health messages also contribute to vaccine hesitancy [16]. Previous studies on vaccination have also focused on vaccine hesitancy [16,17,18,19,20]. These studies have found that the factors influencing people’s reluctance to be vaccinated include the country of origin of vaccines, the technology and certification process of vaccines [17], knowledge about vaccines, beliefs in and experiences with COVID-19 [18], concerns about side effects [19] and vaccine safety [20], the influence of media information [20], as well as the influence of social disadvantages including lower levels of education and poverty [16]. Young people are the priority group for managing COVID-19 transmission and controlling community transmission [12], as they are the most active age group with regard to group activities. There has been no research to investigate vaccine hesitancy among young people with high vaccination rates from a cultural perspective. To resolve this gap, this study analyzes the reasons for vaccine hesitancy and the cultural factors related to it.

Therefore, the purpose of this study is to: (i) explore the factors that influence COVID-19 vaccine hesitancy among young people; and (ii) evaluate the variables that influence the willingness to be vaccinated.

According to the purpose of the study and the previous literature, the hypotheses of this study are:

**Hypothesis 1.**  *The vaccine brand loyalty has a negative impact on the willingness to be vaccinated*.

**Hypothesis 2.**  *The tendency towards collectivism has a positive impact on the willingness to be vaccinated.*

**Hypothesis 3.**  *The degree of disease anxiety has a positive impact on the willingness to be vaccinated*.

**Hypothesis 4.**  *Perceived infectability has a positive impact on the willingness to be vaccinated*.

## 2. Methods

### 2.1. Research Design

This study was conducted during a period of time when the COVID-19 epidemic was comparatively well controlled (1 September 2022–20 October 2022). People in traditional Chinese society conform to the principal of Asian collectivism. Most vaccine hesitant people tend to be young [21,22]. Therefore, this study mainly investigated the psychological status and behavior of COVID-19-vaccine-hesitant young people. The subjects included students from the university of Taiwan. We recruited volunteers aged 18–26 on campus to join the survey. After clearly stating the research purpose, willing participants were sent an online questionnaire. Due to the COVID-19 pandemic, the survey was distributed using online questionnaires only. A power analysis was conducted prior to this study. Together with an α of 0.05, accepting a power of 0.80, and aiming at detecting medium effect size correlations, a sample size of approximately 134 was determined to be required. This study involved a sample of 232 young people (aged 18–26) living in Taiwan. Willingness to participate in the online survey indicated respondents’ consent to their involvement in the study.

### 2.2. Questionnaires

The questionnaire first investigated demographic variables, including nationality, gender, age, vaccination against influenza, chronic diseases, doses of COVID-19 vaccines, and vaccine hesitancy [23]. In addition, one score was used to measure loyalty to the four vaccine brands currently available in Taiwan. One point was given for willingness to be vaccinated by all four vaccine brands, two points for three brands, three points for the two brands, and four points for one brand. Higher scores represented higher vaccine brand loyalty.

This study also measured variables related to vaccine hesitancy, such as collectivism, disease anxiety, and perceived infectability. The items measuring collectivism were modified from those of Triandis and Gelfand (1998) [24], with a total of eight items. This construct was measured using a 9-point scale, ranging from 1—never or definitely no, to 9—always or definitely yes. The items measuring anxiety were modified from those of Yoon et al. (2021) [25], with a total of six items. This construct was measured using a 5-point rating scale, from 1—strongly disagree, to 5—strongly agree. The items measuring perceived infectability were modified from those of Duncan et al. (2009) [26], with a total of four items. This construct was measured using a 5-point rating scale, from 1—strongly disagree, to 5—strongly agree.

First, to test the reliability and validity of the measures, this study first conducted an analysis using Cronbach’s α. If α is 0.60 or greater, it shows good reliability [27]. Next, a confirmatory factor analysis (CFA) was conducted to test composite reliability (CR) and average variance extracted (AVE). Composite reliability is a measure of internal consistency in scale items and the average variance extracted is a measure of the amount of variance that is captured by a construct in relation to the amount of variance due to measurement error. AVE is commonly used to validate constructs. For collectivism, Cronbach’s alpha was 0.946, CR was 0.956, and AVE was 0.729. For anxiety, Cronbach’s alpha was 0.965, CR was 0.972, and AVE was 0.854. For perceived infectability, Cronbach’s alpha was 0.913, CR was 0.940, and AVE was 0.796. In this study, all of the variables’ CR > 0.7 and AVE > 0.5, thus showing that the reliability and validity of this study were acceptable [28].

### 2.3. Data Analysis

This study used the G*Power to compute required sample sizes and the Statistical Package for Social Sciences (IBM SPSS Inc., Chicago, IL, USA) to perform statistical analyses. Average mean, standard deviation, range, and percentages were used in the descriptive statistics. The statistical methods included the chi-square test and the Mann–Whitney U test to find the contribution of the variables and the significance of their effects.

## 3. Results

Two hundred and fifty people were invited to participate in this study. A total of 232 young people were formally included in the study. Among them, 156 were women (67.2%), 76 were men (32.8%), and the average age was 19.9 years (range 18–26). There were 86 Vietnamese students (37.1%) and 146 Taiwanese students (62.9%). According to the survey on willingness to continue being vaccinated against COVID-19, 208 students (89.6%) were willing to be vaccinated and 24 students (10.4%) were unwilling to be vaccinated. Of those who were willing to be vaccinated, 146 were female and 62 were male. There were 10 females and 14 males who were not willing to receive vaccinations. The score test showed that gender was responsible for a statistical difference in willingness to be vaccinated against COVID-19 (*p* = 0.005); the proportion of females willing to be vaccinated was relatively higher. Among those who were willing to receive vaccination, 13 (5.7%) had received one dose, 44 (18.9%) had received two doses, and 175 (75.4%) had received three doses. Therefore, nearly 75% of the young people had received additional vaccine doses. There were 189 (81.4%) participants who had received influenza vaccines, including 129 (68.2%) females and 60 (31.8%) males. There were 43 people who had never received influenza vaccines, 16 males (37.2%) and 27 females (62.8%). While 10 females and 4 males had a history of chronic diseases, only 1 of them was unwilling to receive COVID-19 vaccines. There was also no statistically significant difference between willingness to be vaccinated against COVID-19 and influenza and a history of chronic diseases (*p* = 0.389 vs. *p* = 0.685).

Table 1 showed that, for those who were willing to be vaccinated against COVID-19, the reasons for their willingness were prevention of COVID-19 infection (24.4%), fear of transmitting the virus to family members (15.8%), fear of contracting COVID-19 (15.5%), suggestions from relatives and friends (11.5%), availability of vaccines free of charge (9.9%), persuasion of government and medical staff (9.2%), requirements of work units (8.2%), and requirements of school attendance (5.5%). The top three reasons for women to be vaccinated were the prevention of COVID-19 infection, fear of transmitting COVID-19 to family members, and fear of contracting COVID-19. Furthermore, women were least concerned about the requirements of school attendance. The top three reasons for men to be vaccinated were the prevention of COVID-19 infection, suggestions from friends and relatives, and the fear of transmitting COVID-19 to family members. Men were also least concerned about the requirements of school attendance.

The reasons why young people did not want to be vaccinated against COVID-19 were mainly fear of side effects (36.4%), lack of time (15.9%), being in the non-high-risk group (11.4%), the opinion that they feel so healthy that they do not need to be vaccinated (9.1%), the view that COVID-19 vaccines are ineffective (9.1%), the opinion that injections and taking medicine should be avoided as much as possible (9.1%), the opinion that COVID-19 is not a serious illness (4.5%), and the view that they will not contract COVID-19 (4.5%). Among these, the top two reasons for women’s unwillingness to be vaccinated were fear of side effects and lack of time. The least common reasons were the opinion that COVID-19 is not a serious disease and the view that they will not contract COVID-19. The top two reasons for men’s unwillingness to be vaccinated included fear of side effects and lack of time. The least common reasons were the opinion that COVID-19 is not a serious disease, the view that they will not contract COVID-19, the view that COIVD-19 vaccines are ineffective, and the opinion that injections and taking medicine should be avoided as much as possible.

For all respondents, if they were willing to be vaccinated, the vaccine brands they preferred were Pfizer-BioNTech (BNT) (40.9%), Moderna (27.8%), AstraZeneca (23.4%), and Medigen or other vaccines (7.8%).

A survey of all the participants was conducted with regard to their tendency towards collectivism, degree of disease anxiety, vaccine brand loyalty, and perceived infectability. First, those who were willing to be vaccinated had a higher sense of collectivism than those who were not (6.4 vs. 3.7, *p* < 0.001). Second, in terms of vaccine brand loyalty, those who were willing to be vaccinated had lower brand loyalty than those who were unwilling to be vaccinated (3.2 vs. 3.5, *p* = 0.034). Furthermore, those who wanted to receive vaccination had a higher level of anxiety than those who did not (3.5 vs. 2.3, *p* < 0.001). Finally, although the self-perceived degree of infectability was lower among those who were willing to be vaccinated than among those who were unwilling, there was no statistically significant relationship between willingness to be vaccinated and perceived infectability (*p* = 0.967). See Table 2 for detailed results.

## 4. Discussion

Vaccination is an important strategy to prevent infectious diseases. Vaccination can effectively prevent infection, the risk of severe illness, hospitalization, and death after infection. Complete vaccination against COVID-19 can improve herd immunity [29]. COVID-19 has been prevalent for many years, and in order to promote economic recovery, countries have opened their borders one after another. The large flow of people may cause a large number of infections and aggravate the emergence of virus variants. Based on the reason for disease prevention, improving the continuous vaccination of basic doses and supplementary doses of people in various countries and reducing vaccine hesitancy it is an effective way to reduce the spread of the epidemic. Public health scholars and policy makers often emphasize that vaccination can cause herd immunity. However, under the fatigue of vaccination, it is necessary to investigate their willingness to be vaccinated for different groups, and to formulate a solution that suits their preferences based on their hesitation. In addition, people will hesitate when weighing the risks and benefits of vaccination. Therefore, the greatest contribution of this study is to explore the reasons from a cultural perspective and propose countermeasures to make persuasion work more smoothly.

The results of this study show that the willingness of women to continue with vaccination was higher than for that of men. This finding is different from that of Zintel et al. (2022) [30], who found that women were less willing to be vaccinated against COVID-19 than men, while men were more likely to be vaccinated [30]. However, in the vaccination study on the Asian population by Hawlader et al. (2022) [23], it was shown that female participants in India were more willing to be vaccinated than male participants. This study also showed that women in the young Asian group were more willing to receive vaccination than men.

Our study also illustrates that the main reason for willingness to continue vaccination was not only to prevent the contraction of COVID-19, but also the fear of transmitting the disease to family members. Wei and Li (2021) pointed out in their study that the respondents with relatively higher collectivism values adopted many pandemic prevention behaviors, including following the government’s requirements to wear masks, maintaining social distancing, and sharing information about COVID-19 prevention, in pursuit of the greater good. These respondents believed that their actions not only protected themselves, but also protected others from COVID-19 [31]. The present study also shows a similar trend. The Taiwanese culture emphasizes collectivism; under the influence of such a culture, people with a high level of collectivism protect themselves and their families through continuous vaccination for altruistic reasons. These people do not want their families to be affected by their lack of vaccination. Therefore, they are more willing to comply with the government’s proposal to receive a booster injection.

As for vaccine hesitators, their main concern was that vaccines would cause side effects. The Taiwan Centers for Disease Control has declared that COVID-19 vaccines are safe, with the condition that the expected benefits outweigh the risks. However, reports of adverse events continue to emerge. Therefore, the public still holds doubts about continuous vaccination [32]. It is important to continue educating the public through the media about the fact that the benefits of vaccination outweigh its disadvantages, in order to remove the public’s doubts about the advantages of vaccination. The government can also use the persuasive ability of family and friends to move the public to accept vaccination. Another factor that prevented people from being vaccinated is lack of time. Currently, vaccination has to be booked through the Vaccination Registration and Booking System of counties, cities, and medical institutions. Not every medical institution is allowed to provide COVID-19 vaccines, which leads to a lack of convenience in terms of time and place of vaccination, thus reducing the willingness to be vaccinated. Therefore, if possible, setting up vaccination-as-you-go stations in places where people conduct their daily activities, such as in shopping centers or at public transport stations, may increase the willingness of people to receive vaccination.

It was previously noted that university students in Hong Kong preferred vaccines from overseas rather than those from Hong Kong or China, with vaccines from overseas increasing the willingness to be vaccinated by more than 65% [33]. This finding indicates preferences for certain vaccine brands. In this study, those who were willing to receive continuous vaccination had low brand loyalty and were willing to receive vaccines as long as they were officially approved vaccine brands, while vaccine hesitators had high brand adherence. The government should actively engage in health communication, disseminate and update current experimental data to the public through various channels, and dispel the spread of rumors to increase public acceptance of different vaccine brands.

It was previously noted that the pandemic can cause disease anxiety among the public and lead to a rush for supplies [2]. The current study also shows that young people who were willing to receive continuous vaccination had a higher sense of anxiety than vaccine hesitators. This finding reveals that disease anxiety increases willingness to be vaccinated. The COVID-19 pandemic in Taiwan is still affecting people’s lives. Therefore, the government should continue to advocate that people protect themselves and their families through vaccination in order to reduce disease anxiety.

This study had some limitations. First, there were only four government-approved vaccine brands during the period of the survey, so the vaccine brand selectivity was somewhat limited. Furthermore, this study adopted convenience sampling, and although university students from different countries were included in the sample, the number of vaccine hesitators was small, so the generalization of the study was limited. It is also suggested that a follow-up study could be conducted to compare vaccine hesitancy among people in Asian countries that share collectivist cultures, or among people of different countries and age groups. While this study contributes to research on cultural values, it is recommended that future research should examine the relationship between changes in vaccine hesitancy at each point in time from a longitudinal perspective. Finally, this study was conducted during a period of time when the COVID-19 epidemic was comparatively well controlled, so the situation may be different from the period of the epidemic outbreak.

## 5. Conclusions

Unlike previous studies, this study shows that collectivism is associated with willingness to be vaccinated, as people do so not only to protect themselves, but also to protect their families. Therefore, when promoting COVID-19 prevention policies, the government can make use of altruistic incentives to motivate people to follow pandemic prevention guidelines in order to achieve the goal of herd immunization.

## Figures and Tables

**Table 1:** Survey on continued vaccination willingness.

Variables		Gender No. (%)		Count
		Female Group	Male Group	
Willing to be vaccinated	Fear of contracting COVID-19	62 (68.8%)	28 (31.2%)	90
	Prevention of COVID-19 infection	103 (72.0%)	40 (28.0%)	143
	Persuasion of government and medical staff	35 (64.8%)	19 (35.2%)	54
	Suggestions from relatives and friends	34 (50.7%)	33 (49.3%)	67
	Fear of transmitting the virus to family members	64 (68.8%)	29 (31.2%)	93
	Availability of vaccines free of charge	41 (70.6%)	17 (29.4%)	58
	Requirements of work units	29 (60.4%)	19 (39.6%)	48
	Requirements of school attendance	17 (53.1%)	15 (46.9%)	32
Unwilling to be vaccinated	Part of the non-high risk group	2 (40%)	3 (60%)	5
	COVID-19 is not a serious illness	0 (0%)	2 (100%)	2
	Feeling so healthy that I do not need to be vaccinated	1 (25.0%)	3 (75.0%)	4
	I will not contract COVID-19	0 (0%)	2 (100.0%)	2
	COVID-19 vaccines are ineffective	2 (50.0%)	2 (50.0%)	4
	Fear of side effects	7 (43.8%)	9 (56.2%)	16
	Injections and taking medicine should be avoided as much as possible	2 (50.0%)	2 (50.0%)	4
	Lack of time	3 (42.9%)	4 (57.1%)	7

**Table 2:** The relationship between willingness to be vaccinated and various variables.

Variables	Willing to Be Vaccinated	Mean	Standard Deviation	*p*-Value
Vaccine Brand Loyalty	No	3.5	0.8	0.034 *
	Yes	3.2	0.8	
Disease Anxiety	No	2.3	1.1	<0.001 *
	Yes	3.5	1.0	
Tendency towards Collectivism	No	3.7	1.8	<0.001 *
	Yes	6.4	1.5	
Perceived Infectability	No	3.2	1.3	0.967
	Yes	3.2	1.5	

* *p* < 0.05.

## Data Availability

Not applicable.
